# Socioeconomic differences in dietary habits in Italy before and during COVID-19 pandemic: secondary analysis of a nationwide cross-sectional study

**DOI:** 10.1186/s12889-023-17530-6

**Published:** 2024-01-11

**Authors:** Jacopo Dolcini, Elisa Ponzio, Marcello Mario D’Errico, Pamela Barbadoro

**Affiliations:** 1 Department of Biomedical Sciences and Public Health, Section of Hygiene, Preventive Medicine and Public Health, Polytechnic University of the Marche Region, Ancona, Italy; 2 Centre of Obesity, Marche Polytechnic University, Via Tronto 10a, Ancona, 60126 Italy

**Keywords:** Healthy eating, COVID-19, Socioeconomic inequalities, Food, Public health

## Abstract

**Background:**

Several socioeconomic conditions may influence subjects’ adherence to healthy eating habits. Food consumption may be influenced by external stress during crisis periods; however, the effects of these events on food habits are difficult to predict. Also, a pandemic crisis like the recent COVID-19 pandemic may have influenced dietary habits and food consumption. The objective of this study was to compare the dietary habits of Italian people before the COVID-19 pandemic with those belonging to the year 2020 in a nationwide population sample.

**Materials and methods:**

Information on dietary habits has been obtained from the multi-purpose survey on families’ “Aspects of daily life”, carried out in Italy by the Italian National Statistics Institute (ISTAT). We analyzed data coming from 2016 and 2020 editions of the survey (43,000 subjects each year). We used population attributable fraction (PAF) adjusted for age, defined as the proportional reduction in unhealthy diet that would occur if all participants had had a higher education, assuming higher educated individuals as more socially advantaged. Prevalence association for each dietary exposition has been calculated through logistic regression.

**Results:**

Looking at aggregated data from 2016 and 2020 both men and women showed a high prevalence of unhealthy dietary habits. Regarding men, excessive consumption of eggs, pork meat, and bovine meat was characterized by a PAF attributable to socioeconomic conditions to an extent greater than 30%. Women showed the same trend. Focusing on different years of investigation, in 2020, during the COVID-19 pandemic, men and women increased their consumption of eggs, cooked fats, snacks, and sweets, and reduced consumption of fruits and vegetables. Additionally, women increased the assumption of dietary products and meat. Both sexes registered an increase in overweight and obese subjects in 2020.

**Conclusions:**

To our knowledge, this study was the first in our country to use a yearly, nationwide sample to analyze dietary habits by examining specific types of various foods on a nationwide scale and establishing a correlation between these habits and the COVID-19 pandemic. Our results showed unbalanced dietary habits of the Italian population with an excess of consumption of several foods like eggs, cooked fats snacks, and sweets with low consumption of fruits and vegetables. Socioeconomic differences influence food choices but in a complex way since they seemed to affect some wrong dietary habits but not others, especially regarding fruits and vegetables assumption where differences were less evident among social classes. Outside stressors like a crisis period such as the COVID-19 pandemic seem to have an important role in both men and women regarding the assumption of so-called “junk food”.

## Introduction

Several socioeconomic conditions may influence people’s adherence to healthy eating habits [1, 2]. It has been shown that socioeconomic status may influence the consumption of fruits and vegetables [3], meat [4], as well as milk and dairy foods [5]. These inequalities have been studied among different countries [3, 6]. The process that leads to food choices is complex and it has been studied from several points of view [7–9]. Recently, cultural elements started to gather attention since some studies have shown how educational attainment may play an important role in terms of food habits [10] and research showed how education plays a separate and distinct role in terms of contributions to health outcomes [11–13]. Recently some authors have shown how even in Italy, the homeland of the so-called Mediterranean diet [14, 15], there are conflicting results regarding the actual adherence to this dietary pattern [14, 15]. Moreover, food consumption may be influenced by external stress, particularly during crisis periods; however, the effects of these events on food habits are difficult to predict. It has been shown how Italy’s 2008 economic crisis had ambivalent effects, apparently mitigating social inequalities regarding nutrition patterns and behaviors [16]. Studies concerning possible external influences on food consumption are fundamental to understanding public health complex dynamics; in fact, it has been postulated that in some countries like Italy, the presence of the Mediterranean diet could be one of the reasons for positive health outcomes minimizing the impact of the health inequalities [16, 17]. In this context, a pandemic crisis like the recent COVID-19 pandemic may have influenced dietary habits and food consumption. Some studies have reported an increase in eating and consumption of “junk” food [18] and some retrospective studies have shown how also in Italy, during the”lockdown “ (encompassing the 9th of March to the 3rd of May 2020 period,in Italy), people faced negative changes in lifestyles/eating behaviors with potential negative health impact [19, 20]. However, results are not always in accordance since it has been observed also a major adherence to the Mediterranean diet during the lockdown, especially in younger people [21], or generally healthier habits [22]. Many of the aforementioned works have investigated these changes through surveys administered during the pandemic situation and sometimes on a small sample population.

This study aimed to compare the dietary habits of Italian people before the COVID-19 pandemic with those belonging to the year 2020 in a nationwide population sample, to highlight possible unhealthy behaviors in terms of food consumption that may be increased or not during a pandemic crisis. To our knowledge, the novelty of our study resided in the possibility of having combined important elements such as: Comparing data spanning both pre- and during the COVID-19 crisis obtained from a nationwide and ample population sample, using a survey that is part of the integrated system of multipurpose surveys on families aimed to detect a plurality of behavioral dimensions including food habits, and focusing on the population attributable fraction leveled on the education level.

## Materials and methods

### Sample selection

Information on dietary habits has been obtained from the multi-purpose survey on families “Aspects of daily life”, carried out in Italy by the Italian National Statistics Institute (ISTAT) [23]. We analyzed data coming from 2016 and data coming from the 2020 editions of the survey, publicly available from the ISTAT data warehouse. The survey is carried out on a yearly basis, in a representative sample of the Italian population of about 43,000 subjects and is part of the integrated system of multipurpose surveys on families aimed to detect a plurality of behavioral dimensions and segments of daily life. The questionnaire is standardized, and it is administered both in face-to-face mode by trained interviewers and in self-compilation. In the specific case, the two populations (2016 vs 2020) for the current surveys, have been identified by the Italian National Statistics Institute within the set of municipalities which has been divided into two subsets: Municipalities with larger demographic size constitute a separate stratum and are defined as Self-Representative (SR); the remaining municipalities are defined as Non Self-Representative (NSR) and are divided, based on demographic size, into strata of equal breadth. From these strata, the sample municipalities (two for each stratum) were selected with probabilities proportional to their size. For each of the municipalities involved in the survey (SR and NSR), a cluster sampling is carried out: the clusters—the families—are randomly selected from the registry list, and all the members belonging to the actual family are surveyed. The minimum number of sample families for each municipality has been set at 24. The families are selected for each sample municipality from the theoretical sample selected for the Master Sample; for each family included in the sample, the characteristics under investigation of all actual members belonging to the same family are recorded. The size of the theoretical sample in terms of families, set at the national level primarily based on cost and operational criteria, is approximately 24,000 families. The number of involved sample municipalities should not exceed 900, to allow for effective control and supervision. The allocation of the sample of families and municipalities, among the various regions, has therefore been calculated by adopting a compromise criterion to ensure both the reliability of estimates at the national level and that of estimates within each of the territorial domains [23].

In this study, we focused on those questions regarding dietary habits with particular attention to the assumption of specific kinds of food (Table 1).

**Table 1:** Short definition, description, and operative definition of used variables to assess dietary habits

Short definition	Definition
Too much bovine meat	Excessive assumption of bovine meat 1 or more times each day
Too many carbohydrates	Assumption of bread, pasta, rice 1 or more times a day together with potato consumption 1 or more times a day
Few vegetables	Assumption of large leafy vegetables raw or cooked once a day or less
Few fruits	Assumption of fruits once a day or less
No “5 a day”	No assumption of 5 portions of fruits or vegetables each day according to OMS criteria
No “3 a day”	No assumption of 3 portions of fruits or vegetables each day
Too much white meat	Assumption of chicken once or more each day
Too much pork meat	Excessive assumption of pork meat 1 or more times each day
Too much cured meat	Assumption of cured mead once or more each day
Too much meat	Assumption of meat once or more each day
Too much meat not considering cured meat	Assumption of meat once or more each day excluding cured meat
Few fish	Assumption of fish less than a few times a week or never
Few legumes	Assumption of legumes less than a few times a week or never
Too much dairy products	Assumption of dairy products 1 or more times a day
Too many eggs	Assumption of eggs 1 or more times a day
Few proteins	Low intake of protein foods (meat, fish, legumes, dairy products, eggs)
Too much raw fats	Frequent use of butter, lard, or other vegetable fats and oils (seeds. margarine) for raw dressing
Too much cooked fats	Frequent use of butter, lard, or other vegetable fats and oils (seeds, margarine) for cooking
Too many snacks	Assumption of snacks a few times a week or more
Too much salt	Low attention to salt intake (added or already present in food)
Overweight	Body mass index > 25
Obese	Body mass index > 30
No breakfast	Absence of habit of having breakfast
Too many sweets	Assumption of sweets sometimes a week or more

We reclassified variables on dietary lifestyles for each kind of food and food group based on their nutritional properties, dichotomizing in appropriate/inappropriate behaviors considering the frequency of assumption through definitions coming from the scientific literature [24–26] and through comparison and review by nutrition and food hygiene experts done in previous works [16].

### Statistical analysis

To appropriately account for socioeconomic inequalities, we used population attributable fraction (PAF) adjusted for age, defined as the proportional reduction in unhealthy diet that would occur if all participants had had a higher education, assuming higher educated individuals as more socially advantaged [16]. PAF has been defined as the proportion of cases for an outcome of interest that can be attributed to a given risk factor among the entire population [27, 28]. Specifically, PAF can be defined as:

$$PAF=\frac{\mathrm{Incidence}\;\mathrm{rate}\;\mathrm{imn}\;\mathrm{total}\;\mathrm{population}-\mathrm{Incidence}\;\mathrm{rate}\;\mathrm{unexposed}}{\mathrm{Incidence}\;\mathrm{rate}\;\mathrm{in}\;\mathrm{total}\;\mathrm{population}}$$

The above equation could also be expressed as either Levin’s or Miettinen’s formula [29] that we used for our data:

$$PAF=\frac{p\;\left(RR-1\right)}{p\;\left(RR-1\right)+1}$$

where p is the prevalence of the risk factor and RR is the relative risk of incidence of the disease of the exposed over the non-exposed.

We obtained positive PAFs when less educated people had a greater consumption compared to more educated ones, and negative PAFs in the opposite case. To account for level of instruction, we recoded educational level into four classes (more than 13 years of study, 8–13 years of study, 5–8 years of study, less than 5 years of study). We analyzed data from subjects 20 years old and older; age has been subsequently stratified according to sex into classes:20–34 years, 35–54 years, 55–64 years, and 65 years or older. We also stratified the population according to BMI focusing our attention on overweight (25 ≤ BMI ≤ 29.99) and obese people (BMI ≥ 30). Age class variations compared to all Italian population values, have been standardized through z scores, and classified according to the number of deviations. Z score was intended as the number of standard deviations from the mean values of the Italian population. We first analyzed aggregated data from 2016 and 2020, then we compared relative variation between the two years. Prevalence association for each dietary exposition has been calculated through logistic regression and the level of confidence has been set at 95%.

## Results

Baseline characteristics of our study populations are shown in Table 2, according to year of interview and stratified for age class, marital status, and education level.

**Table 2:** Baseline characteristics

Variable	Characteristics	Year 2016 (%)	Year 2020 (%)
**Sex**	**Male**	20,971 (48.36)	20,518 (47.93)
	**Female**	22,389 (51.64)	22,292 (52.07)
**Age classes**	** < 20**	7,793 (17.97)	7,302 (17.06)
	**20–34**	6,532 (15.06)	6,017 (14.06)
	**35–54**	13,025 (30.04)	12,012 (28.06)
	**55–64**	5,717 (13.18)	6,533 (15.26)
	** > 65**	10,293 (23.74)	10,946 (25.57)
**Marital Status**	**Single**	15,366 (35.44)	15,402 (35.98)
	**Married/cohabitant**	19,172 (44.22)	18,374 (42.92)
	**Divorced**	3,164 (7.30)	3,149 (7.36)
	**Widow/Widower**	3,532 (8.15)	3,632 (8.48)
	**Not available**	2,126 (4.9)	1,689 (3.95)
**Education Level**	**> 13 years of study**	5,112 (11.79)	6,177 (14.43)
	**8 to 13 years of study**	13,692(31.58)	13,970 (32.63)
	**5 to 7 years of study**	11,919 (27.49)	11,351 (26.51)
	**< 5 years of study**	10,511 (24.24)	8,682 (20.28)
	**Not available**	2,126 (4.9)	2,630 (83.95)

**Fig. 1:**
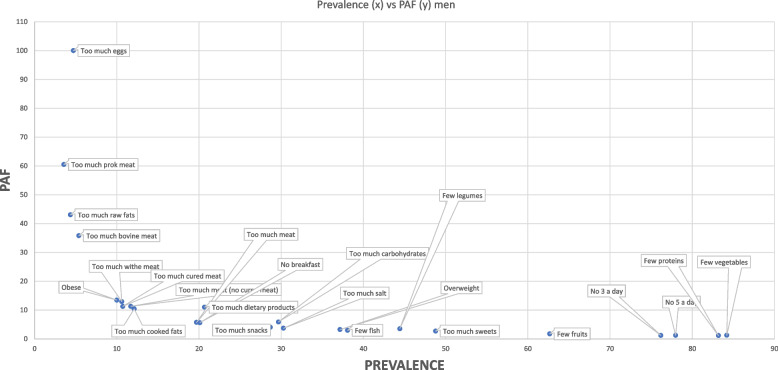
Prevalences and PAFs aggregated data 2016 and 2020 men

**Fig. 2:**
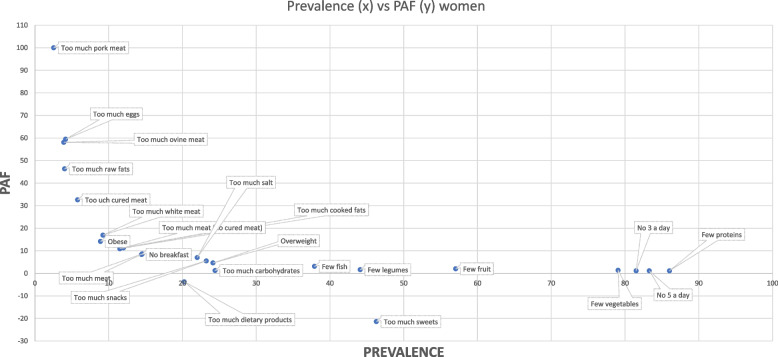
Prevalences and PAFs aggregated data 2016 and 2020 women

Of a total of 86,170 subjects coming from 2016 and 2020, 1,095 were younger than 20 years old. So, our analyses have been performed on a remnant of 71,075 subjects divided into 33,724 males and 37,351 females. As we can see from Figs. 1 and 2 both men and women showed a high prevalence of unhealthy dietary habits like few vegetables assumption (M = 84.1% *N* = 28,363; *F* = 78.3% *N* = 29,258), few fruits assumption (M62.8% *N* = 21,167; *F* = 56.4% *N* = 21,074) and few proteins intake (M = 84.9% *N* = 28,626; *F* = 87.9% *N* = 32,829). Both men and women had a high prevalence of no assumption of five portions (M = 81.5% *N* = 27,480; *F* = 86.8% *N* = 32,405) or at least three portions (M = 79.7% *N* = 26,878; *F* = 84.9% *N* = 31,726) of vegetables and fruits each day. Another aspect to highlight is that socioeconomic features seemed to influence unhealthy habits of food assumption. Regarding men, excessive consumption of eggs, pork meat, and bovine meat was characterized by a PAF attributable to socioeconomic conditions in an extent greater than 30%. Women exhibited a similar trend, as foods with the lowest prevalence in the population were characterized by a greater attributable fraction of PAF due to socioeconomic differences. This is the case of excessive assumption of pork meat, eggs, ovine meat, raw fats, and cured meat representing more than 30% of PAF. Interestingly, women also demonstrated negative PAFs associated with excessive intake of dairy products and increased consumption of sugar-based products. This implies that, in this scenario, lower socioeconomic conditions were linked to a reduced consumption of these two types of foods. Analyzing differences among age classes (Table 3), some unhealthy dietary habits (such as few consumptions of proteins and not having breakfast) were present both in men and women all among age classes. Low consumption of fruits and vegetables is particularly frequent with advancing age in both men and women, as is the prevalence of overweight and obese individuals. Again, even if the prevalence of consumption of different kinds of foods is comparable among age classes, PAF assumed values quite far from standard deviation like the amount of cooked fats assumption, sweets assumption, salt consumption, and obese PAF which is higher in younger people in both men and women.

**Table 3:** Variations in z-score ^a^ compared to the Italian average of prevalence and fraction attributable to social inequalities (PAF), ^b^by age class. Aggregated years 2016–2020

	Too much carboidrates		No 3 a day		Too much meat		Few fish		Few legumes		Few proteins		Too much cooked fats		Too much sweets		Too much salt		Overweight		Obese		No br eakfast	
	PREV	Paf %	PREV	Paf %	PREV	Paf	PREV	Paf %	PREV	Paf %	PREV	Paf %	PREV	Paf %	PREV	Paf	PREV	Paf %	PREV	Paf %	PREV	Paf %	PREV	Paf %
Men																								
20–34	0	--	-	+	++	-	+	+	++	+++	-	---	+	+++	+	+++	++	+++	-	++	–	+++	+	-
35–54	---	--	0	0	0	0	+	--	0	0	+	0	++	+++	0	+++	0	+++	+	-	0	0	++	-
55–64	---	0	+	--	-	+	0	-	-	0	++	0	-	+++	-	+++	-	+++	++	-	++	0	++	-
≥ 65	---	---	+++	--	-	++	--	+	--	++	+++	0	-	+++	---	0	--	+++	++	0	++	0	-	++
All age classes	29,68	21,37	76,19	1,24	19,69	5,80	38,07	2,95	44,43	1,29	82,94	1,15	12,11	6,21	48,77	-1,95	30,29	-1,43	37,15	3,11	9,99	12,96	20,10	5, 64
Women																								
20–34	-	0	-	0	++	-	+	++	0	---	+++	0	0	+++	+	+	++	0	-	++	-	+++	+	0
35–54	-	0	0	0	0	0	0	0	-	0	+++	0	0	+++	0	--	0	0	0	0	0	0	++	-
55–64	--	+++	++	-	--	++	--	0	---	0	+++	+++	---	+++	-	++	-	0	+	-	+	0	++	0
≥ 65	++	+	+++	---	-	0	0	--	0	0	+++	0	+	+++	--	0	--	+++	++	-	++	0	-	+++
All age classes	24,44	1,73	81,52	1,14	14,56	9,10	37,91	3,12	44,10	1,45	86,04	1,11	11,52	6,30	46,29	1,90	22,01	3,54	24,14	4,50	8,89	13,65	14,46	8, 62

^a^The changes in z-scores have been reclassified according to the following qualitative criterion: ---(z ≤ -1.5), -(-1.5 < z ≤ -1); -(-1 < z ≤ -0.5); 0 (-0.5 < z < 0.5); + (0.5 ≤ z < 1); +  + (1 ≤ z < 1.5); +  +  + (z ≥ 1.5)

^b^The attributable reference fractions were calculated considering the most educated as the reference category

**Fig. 3:**
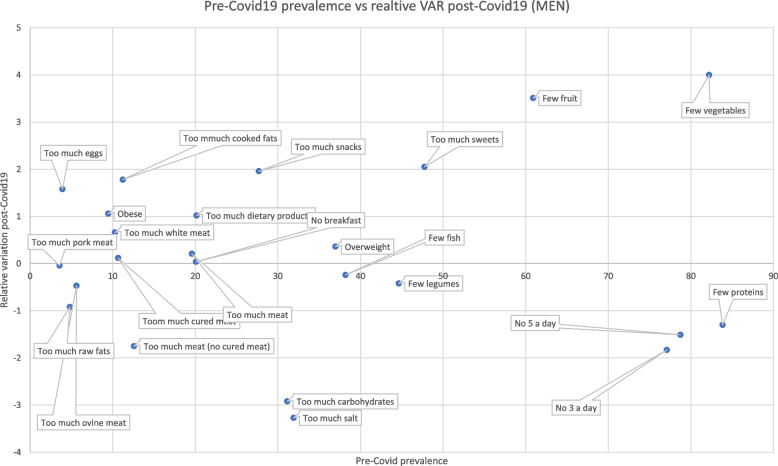
Pre Covid19 prevalences and relative variation (VAR) post-Covid19 (men)

**Fig. 4:**
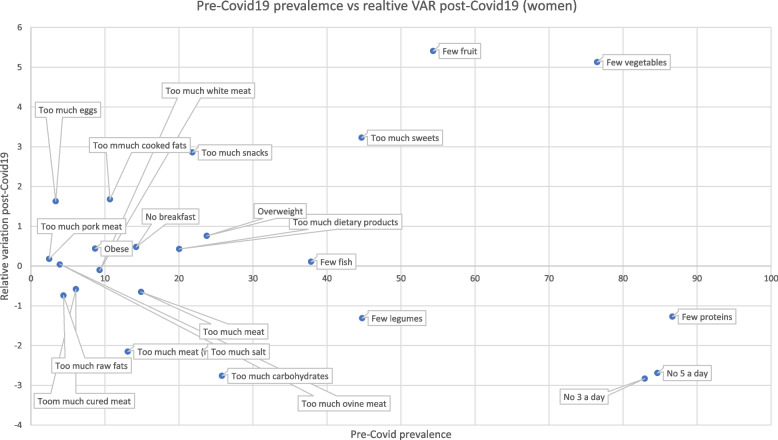
Pre Covid19 prevalences and relative variation (VAR) post-Covid19 (women)

After considering aggregated data, we analyzed the prevalence variation of different food consumption comparing prevalence levels in 2016 before the COVID-19 pandemic and their relative variation with values registered in 2020 during the COVID-19 pandemic (Figs. 3 and 4). Both men and women showed unhealthy profiles of food consumption since men increased their consumption of eggs, cooked fats, snacks, and sweets, and reduced consumption of fruits and vegetables. Moreover, compared to the 2016 edition of the survey there has been an increase in obese and overweight men in 2020. Regarding women, they also showed an increased assumption of eggs, cooked fats, snacks, sweets, dietary products, meat, and a reduced intake of fruits and vegetables. As seen in men, women in 2020 registered an increase in overweight and obese subjects. Simultaneously, positive habits have been noted in both men and women, marked by a decrease in the percentage of individuals with low legume and protein intake, reduced consumption of raw fats, cured meats, carbohydrates, salt, and ovine meat, as well as a decline in the number of people not meeting the daily recommended intake of 5 or 3 portions of fruits and vegetables.

## Discussion

The data presented in our study requires careful consideration regarding the dietary habits of the Italian population. The aggregated data from 2016 and 2020, indicated that socioeconomic disparities might have played a role in shaping food consumption patterns. Notably, it is intriguing to observe that the most common unhealthy habits, such as limited intake of vegetables, fruits, and proteins among both men and women, exhibited the lowest values of Population Attributable Fraction (PAF) attributable to socioeconomic differences. This suggests that these nutritional aspects are widely accepted across various social classes, even though they may not be healthy. These data are in accordance with previous study findings, which reported a general decline in Mediterranean diet adherence characterized by great assumption of fruits and vegetables [30]. One of the reasons may reside in the increased cost of low-energy density foods, like fruits and vegetables [31, 32]. It has been shown how energy-dense foods like meat, fats, and in general high-calorie food are less expensive than lower energy foods [2, 31, 33]. However, results regarding the inverse relationship between food price and energy density are still under debate [32, 34]. Examining consumption patterns, our study revealed the prevalence of wrong dietary habits in the Italian population, including high intake of meat, fats, and dairy products. While these habits exhibit prevalence rates ranging from 10 to 40% in both women and men, it’s essential to consider that variables are defined as excessive consumption of specific foods such as consumption of meat once or more a day, assumption of carbohydrates once or more a day, dairy products assumed once or more a day, and so forth. This could have implied that, even if the improper consumption of a single type of food had a low prevalence, there could have been a cumulative effect of various unhealthy habits, leading to an overall unbalanced diet. This can be testified by the fact that more than 80% of men and women declared insufficient intake of protein, while more than 50% of both sexes declared an excessive assumption of sweet food. A positive aspect emerges when considering certain unhealthy habits mentioned earlier: many of them were influenced by socioeconomic conditions. The Population Attributable Fraction (PAF) was notably high for men in the excessive consumption of eggs, pork meat, raw fats, and bovine meat. For women, it peaked in the consumption of pork meat, eggs, raw fats, and cured meat. These data, when coupled with the previous findings on the low prevalence of fruits and vegetable consumption not influenced by socioeconomic factors,—stratified among different cultural levels in our study—may underscore the significance of educational levels in comprehending unhealthy dietary habits. Simultaneously, an economic factor appeared to mitigate differences among social classes, particularly for certain types of foods.

But another reason may have contributed to our findings: looking at the same data separately and comparing prevalence levels of 2016 and 2020 and their relative variation, we found out how during COVID-19 pandemic there has been an important increase in unhealthy habits in both men and women like excessive consumption of eggs, cooked fats, snacks, and sweets with low consumption of fruits and vegetable. Moreover, in both sexes, there has been an increase in overweight and obese subjects. These results align with findings from other studies conducted globally [17, 35]. Various explanations can account for this, as during pandemic situations, individuals might have favored fast food or instant food products. Moreover, people staying at home tended to consume meals and snacks more frequently [36]. Moreover, depression and anxiety moods that were caused by COVID-19 spreading among all countries, especially during the first wave, may have influenced food habits since these emotional reactions may have increased craving for comfort eating characterized by assumption of high-fat, high sugar or high-calorie food [17, 22, 35]. Further consideration must be done regarding financial hardship, since the COVID-19 pandemic has led to an economic crisis that influenced food behavior, habits, and food insecurity as indicated by several studies [36–38]. This resulted in a heightened likelihood of unhealthy behaviors and choices.

To our knowledge, this study was the first in our country to use a validated yearly questionnaire to analyze dietary habits, examining various types of foods on a nationwide scale and in relation to the COVID-19 pandemic. Our sample was both large and representative of the Italian population, thanks to the study’s design conducted by the National Institute of Statistics (ISTAT). Nevertheless, some limitations should be acknowledged. Given the nature of this study, as a prevalence study rather than a longitudinal one, certain details regarding individuals’ dietary habits might lack precision. This is due to the fact that during interviews, individuals tend to report their most recent habits, which may not necessarily reflect the overall dietary situation throughout the entire year. According to the nature of the survey, all data must be considered declarative including height and weight used to calculate BMI, generating a possible declarative bias in our sample. Moreover, we did not have health information on our subjects so we could not adjust to some pathological conditions like diabetes and hypertension, that can influence food assumption. Lastly, it’s important to note that the official language of ISTAT, the government institution conducting the survey, is Italian. The survey administration was not declared in other languages, which may have resulted in a selection bias. This bias could have excluded individuals, even residents in Italy, who did not fully understand the Italian language.

Taken together, our results showed unbalanced dietary habits of the Italian population: some of them are more represented in some specific age classes but they can be found all along the population. Socioeconomic differences influenced food choices but in a complex way, since they seemed to affect some wrong dietary habits but not others, especially regarding fruits and vegetables assumption where differences were less evident among social classes. Moreover, outside stressors, like a crisis period such as the COVID-19 pandemic, seemed to have an important role in both men and women regarding the assumption of so-called “junk food”, characterized by intake of high calories and high sugars meals.

More studies are needed to better clarify the role of specific socioeconomic conditions on food habits, and how and if a specific variable may affect the assumption of one food rather than another. Furthermore, there is also an urgent need to understand the long-term effects of an important crisis event like COVID-19 on the health habits of people, with particular attention to their food behaviors and BMI and if eventual wrong outcomes, acquired during a pandemic, are easily reversible or not, requiring a lot of education and attention by health policymakers with dedicate prevention campaigns and sensibilization programs.

## Data Availability

The datasets used and/or analyzed during the current study are available from the corresponding author upon reasonable request.
